# Data linkage errors in hospital administrative data when applying a pseudonymisation algorithm to paediatric intensive care records

**DOI:** 10.1136/bmjopen-2015-008118

**Published:** 2015-08-21

**Authors:** Gareth Hagger-Johnson, Katie Harron, Tom Fleming, Ruth Gilbert, Harvey Goldstein, Rebecca Landy, Roger C Parslow

**Affiliations:** 1 Centre for Paediatric Epidemiology and Biostatistics, UCL Institute of Child Health, London, UK; 2 Department of Epidemiology and Public Health, UCL, London, UK; 3 Division of Epidemiology and Biostatistics, Leeds Institute of Cardiovascular and Metabolic Medicine, School of Medicine, University of Leeds, Leeds, UK; 4 Centre for Multilevel Modelling, University of Bristol, Bristol, UK; 5 Centre for Cancer Prevention, Wolfson Institute of Preventive Medicine, Queen Mary University of London

**Keywords:** STATISTICS & RESEARCH METHODS, EPIDEMIOLOGY, data linkage

## Abstract

**Objectives:**

Our aim was to estimate the rate of data linkage error in Hospital Episode Statistics (HES) by testing the HESID pseudoanonymisation algorithm against a reference standard, in a national registry of paediatric intensive care records.

**Setting:**

The Paediatric Intensive Care Audit Network (PICANet) database, covering 33 paediatric intensive care units in England, Scotland and Wales.

**Participants:**

Data from infants and young people aged 0–19 years admitted between 1 January 2004 and 21 February 2014.

**Primary and secondary outcome measures:**

PICANet admission records were classified as matches (records belonging to the same patient who had been readmitted) or non-matches (records belonging to different patients) after applying the HESID algorithm to PICANet records. False-match and missed-match rates were calculated by comparing results of the HESID algorithm with the reference standard PICANet ID. The effect of linkage errors on readmission rate was evaluated.

**Results:**

Of 166 406 admissions, 88 596 were true matches (where the same patient had been readmitted). The HESID pseudonymisation algorithm produced few false matches (n=176/77 810; 0.2%) but a larger proportion of missed matches (n=3609/88 596; 4.1%). The true readmission rate was underestimated by 3.8% due to linkage errors. Patients who were younger, male, from Asian/Black/Other ethnic groups (vs White) were more likely to experience a false match. Missed matches were more common for younger patients, for Asian/Black/Other ethnic groups (vs White) and for patients whose records had missing data.

**Conclusions:**

The deterministic algorithm used to link all episodes of hospital care for the same patient in England has a high missed match rate which underestimates the true readmission rate and will produce biased analyses. To reduce linkage error, pseudoanonymisation algorithms need to be validated against good quality reference standards. Pseudonymisation of data ‘at source’ does not itself address errors in patient identifiers and the impact these errors have on data linkage.

**Strengths and limitations of this study**

- This is the first study to evaluate the rate of data linkage error in the Hospital Episode Statistics (HES) pseudonymisation algorithm.
- The HESID pseudonymisation algorithm is applied to a reference standard clinical data set, the Paediatric Intensive Care Audit Network (PICANet).
- When applied to PICANet data, HESID produces a false match rate of 0.2% (higher for younger patients, males and ethnic minority groups) and a missed match rate of 4.1% (higher for younger patients, ethnic minorities and those with missing data) that underestimates the readmission rate by 3.8%.
- Results may not generalise beyond paediatric intensive care settings.
- Data linkage error in HES itself may be higher, because data quality is lower in HES than in PICANet.

## Introduction

To allow analysis of patients use of healthcare across hospitals and over time, a data resource needs to link together episodes of hospital care that belong to the same person.^1^ In England, Hospital Episode Statistics (HES) are a data set containing data on admissions, outpatient appointments and Accident and Emergency attendances at National Health Service (NHS) hospitals in England.^1^ Each record is submitted to the Health and Social Care Information Centre (HSCIC), who use the HESID pseudonymisation algorithm^2^ to identify all hospital records that should be linked together across the NHS in England, using a range of patient identifiers commonly used internationally in administrative data (eg, date of birth, sex, postcode, ID number).^2^ HES are then released to researchers, with patient identifiers removed. The data are then considered pseudonymised, because the risk of identification has been minimised, although not removed. Researchers assume that hospital episodes with the same HESID refer to the same patient.^2^ Concerns have been raised that implausible clinical scenarios indicate problems with data quality, which can be compounded by the linkage algorithm (eg, a patient dies but is then apparently readmitted).^3^ ^4^ These data are used widely and yet the extent of data linkage error in HES has undergone no investigation against an external reference standard.

Data linkage errors occur when algorithms apply the same ID to more than one patient (a false match) or when different IDs are applied to the same patient (a missed match).^5^ ^6^ Data linkage error has obvious clinical implications relating to safety and confidentiality^7–9^ and is known to introduce bias into statistical analysis. For example, prevalence rates can be under-estimated due to missed matches.^10^ Relative risks can be biased and the direction of effects even reversed,^11^ as linkage success is better for healthier subgroups of the population. Commentators have highlighted the importance of evaluating the extent of linkage error prior to analysis.^12^ To estimate the rate of data linkage error, an independent reference standard is needed that identifies each patient correctly.^13^ No such reference standard is currently available for HESID. Although the algorithm was designed to minimise false matches, it has undergone no evaluation to estimate either the false or the missed match rate. The aim of the current study was to estimate for the first time, the rate of data linkage errors that would be expected in HES when the HES pseudonymisation algorithm is used to link the same patients in a longitudinal hospital record. To highlight the potential impact of these linkage errors on outcome measurement, we identified patient groups most affected by linkage error.

## Methods

To identify linkage errors, we used a reference standard clinical data set with an independently allocated patient ID. To identify causes of linkage errors, we used the patient identifiers in the reference standard data. These are the same patient identifiers used by the HES pseudonymisation algorithm.

### Reference standard: PICANet Patient Identification Number

The hospital data were drawn from the Paediatric Intensive Care Audit Network (PICANet) database for 33 paediatric intensive care units in England, Scotland and Wales (1 January 2004 to 21 February 2014). Since 2002, PICANet has collected data on admissions to paediatric intensive care units (PICUs) in the UK and Ireland.^14^ ^15^ Data are entered by dedicated staff, independent from the main hospital systems, including the same patient identifiers used in the HES pseudoanonymisation algorithm.

We used the PICANet Patient Identification Number (PICANet ID) as the reference standard defining same and different patients across multiple admissions over time because it has a high level of accuracy, patient data are reviewed in audits and by manual review and there are high levels of completeness for key fields such as sex (100%), date of birth (100%) and postcode (96%). Completeness of these fields in HES is not published by the HSCIC routinely, but their data cleaning and extraction rules would suggest that sex and date of birth are close to 100% complete. Postcodes were missing for all birth episodes in 2011/2012 and missing for 2.8% of newborns readmitted in 2011/2012.^4^ In PICANet, systematic validation of NHS number, date of birth, postcode (using the AFD tool: http://www.afd.co.uk) and checks for missing or incongruous values are carried out via a custom designed web data entry interface that is accessed via a highly secure and restricted login. All data processing takes place on the PICANet secure server. Review of the completeness and accuracy of records is carried out by Paediatric Intensive Care Unit staff. This enabled us to compare what would happen to patient records in PICANet if they were pseudonymised by the HES algorithm. Additionally, because PICANet has available the same identifiers as used by the HES algorithm, we could identify which patient identifiers caused data linkage errors following pseudonymisation.

The PICANet PatientID is allocated using a range of identifiers using a three-step probabilistic matching algorithm, by the PICANet team: (1) Weights computed separately for agreement and disagreement on each identifier (date of birth, surname, forename, NHS number, local patient identifier, postcode, sex) are summed, and the highest scoring pair retained; (2) the pair are classified as a non-match, possible match or definite match; (3) possible matches are manually reviewed to estimate match status. Missing data on identifiers is permitted by assigning a zero weight. Unlike with the HESID algorithm, the PICANet ID allows NHS number to differ and still produce a match, if other identifiers agree and produce a sufficiently high probability.

### Data linkage algorithm: HESID

After data from the Secondary Use Service (SUS) are submitted to the Health and Social Care Information Centre (HSCIC) and have undergone data cleaning,^16^ HSCIC apply the pseudonymisation algorithm to link hospital records belonging to the same patient together, on the basis of deterministic linking on patient identifiers at three steps: (1) match on sex, date of birth, National Health Service (NHS) number; (2) match on sex, date of birth, local patient identification number within hospital and postcode; (3) match on sex, date of birth and postcode (excluding communal postcodes or records with a different NHS number).^2^ This data linkage algorithm is designed to minimise the number of false matches, although it has undergone no formal evaluation until now. Patient identifiers are removed before data are released to researchers, who use the pseudonymised HESID to identify records belonging to the same patient. We applied the HES pseudonymisation algorithm to the same identifiers in the PICANet data set and compared them with same and different patients defined by the PICANet PatientID.

### Ethical approval

Collection of personally identifiable data has been approved by the Patient Information Advisory Group (now the NHS Health Research Authority Confidentiality Advisory Group) http://www.hra.nhs.uk/documents/2015/05/piag-register-8.xls. We applied the three-step HESID algorithm according to the rules described in the publicly available documents (V.2)^2^ ^17^ using Stata V.12.1 in order to assign a HESID to PICANet records. This assigned the same HESID to two records if sex and date of birth matched and any one of the following three scenarios applied (a deterministic algorithm): (1) same NHS number, (2) same local ID within hospital and same postcode, (3) same postcode (unless NHS number differs), excluding communal postcodes. We then compared the results of the HESID algorithm with the results of the probabilistic PICANet algorithm. Treating PICANet ID as a gold standard, we then calculated the proportion of true matches, false matches, missed matches and true non-matches. Next, we examined the different scenarios that contributed to linkage success and linkage error, by counting the number of combinations of each identifier pair that could occur.

For linked records (true or false matches), there were 81 possible outcomes of each of the four identifiers being the same as each other, different from each other, or missing (since there were four identifiers and three possible outcomes; 3^4^=81). For missed matches, this generated 324 possible scenarios because there were an additional two identifiers (sex and date of birth) with two possible values (2^2^×3^4^=324).

Age was calculated from date of birth and date of first admission. Estimated or partly anonymised dates of birth were set to missing,^18^ since these would be considered missing by the HESID algorithm. Sex was coded as male, female or missing. Ethnic group was classified as White, Mixed, Asian, Black, Other or Missing.^19^ NHS numbers were considered valid if they passed the standard Modulus 11 algorithm, were 10 digits long, did not have 10 identical digits and were not 1234567890.^17^ Area-based socioeconomic status was derived from the Index of Multiple Deprivation (IMD2010) score, a comprehensive summary of 38 markers of local socioeconomic deprivation across seven domains.^20^ Admissions were classified as planned or unplanned. The number of admissions for each hospital was treated as a proxy for hospital size. Hospital ID and local ID number (both complete within PICANet) were used to represent provider and local ID.

For the main analysis, multilevel logistic regression with the maximum likelihood estimator in Stata V.12.1 was used to identify patient characteristics associated with false matches (vs true matches) and in a separate model, characteristics associated with missed matches (vs true non-matches; see online supplementary appendix 1). The second level of the model acknowledged that patients were nested within different hospitals. Predictor variables were age, sex, ethnic group (Mixed/Asian/Black/Other/Missing vsvs White), unplanned admissions (vs planned), tertile of socioeconomic deprivation (middle vs low, high vs low), missing data on socioeconomic deprivation (typically due to missing postcode), unit size (small/medium/large according to the number of records). We also tested for interactions between ethnicity and deprivation.

In a supplementary analysis, we re-ran the analysis allowing partial matching on date of birth at step 1 of the HES algorithm,^2^ to see if this rule influenced the rate of linkage errors. Date of birth accuracy in PICANet is relatively high however, so we evaluated the impact of replacing 1%, 2% or 5% of the date of birth values as missing and/or transposed (eg, dd/mm to mm/dd, permitted by the HESID algorithm) on linkage success. We also compared estimated to actual readmission rates for different patient groups. We also compared three different approaches to postcode validation (most strict, a balanced approach, most relaxed), in order to evaluate whether data linkage errors were sensitive to postcodes validation rules, ranging from most strict (postcode area, postcode district, space, postal sector, unit code) to most relaxed (spaces removed).

**Figure 1:**
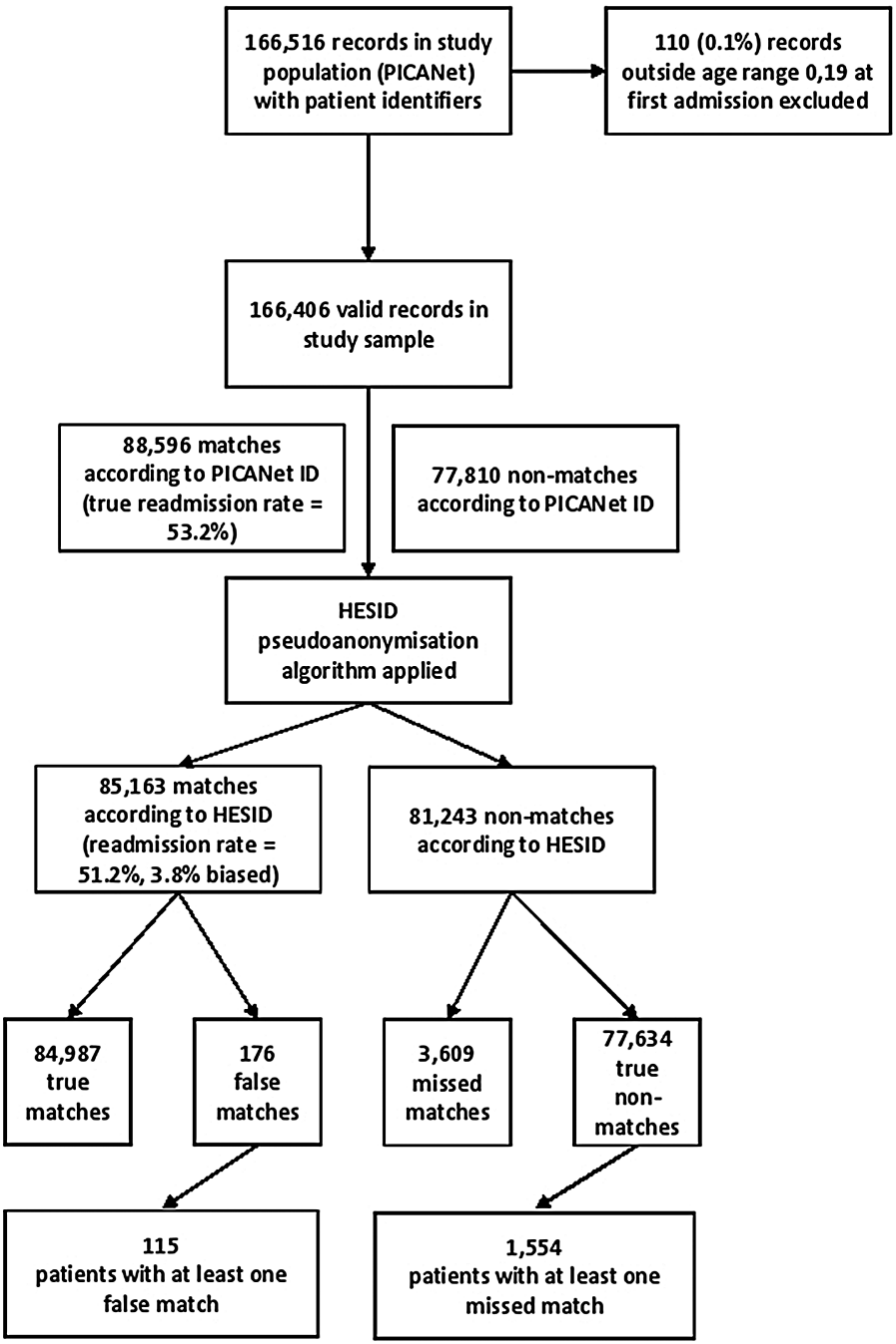
Data linkage errors following application of the Hospital Episode Statistics ID (HESID) algorithm to Paediatric Intensive Care Audit Network (PICANet) records.

As shown in figure 1, the study population comprised 166 516 records from PICANet. After excluding 110 (0.1%) records outside the age range 0–19 at first admission, 166 406 were available for analysis. According to PICANet ID, 88 596 (53.2%) of records were true matches (had been readmitted during the period of data capture 1 January 2004 to 21 February 2014). Using the deterministic HES pseudonymisation algorithm used to link unique patients over multiple episodes of care to create the HESID, 85 163 (51.2%) were matches, showing the HESID underestimated the true readmission rate by 3.8% (100×((53.2%–51.2%)/53.2%)); a risk difference of 2%. Of the 85 163 matches according to HESID, 176 were false matches (0.2% of 77 810 true non-matches; 0.1% of all records). Of the 81 243 non-matches according to the HESID pseudonymisation algorithm, 3609 were missed matches (4.1% of 88 596 true matches; 2.2% of all records).

Table 1 shows the different combinations of identifier values that contributed to false matching. The largest proportion of false matches involved NHS numbers that were missing (n=131, 74.4%) and at least one other identifier differed. There were 38 (21.6%) records that falsely matched even when the NHS number was the same and at least one other identifier differed. The remaining 7 (5%) false matches occurred for other reasons.

**Table 1:** Scenarios involving different combinations of patient identifiers that resulted in false matches (n=176, 0.1%)

Sex (100% valid)	Date of birth (100% valid)	NHS number (58.5% valid)	Hospital (100% valid)	Local ID (100% valid)	Postcode (98.3% valid)	False matches n (%)	
Scenarios involving NHS numbers that differ							
1	1	0*	0	0	0	20 (11.8)	
1	1	0*	0	0	1	70 (41.4)	
1	1	0*	1	0	0	1 (0.6)	
1	1	0*	1	0	1	39 (23.8)	
1	1	0*	1	1	1	1 (0.6)	=131 (74.4%)
Scenarios involving NHS numbers that match							
1	1	1	0	0	0	33 (19.5)	
1	1	1	1	0	0	4 (2.4)	
1	1	1	1	1	0	1 (0.6)	=38 (21.6%)
Other scenarios†						7 (4.0)	=7 (5%)

1=identifier is the same, 0=identifier is different or missing.

*Records with missing NHS numbers are linked by the HESID algorithm, unless a different NHS number is present.

†These scenarios occur when a record has an NHS number (and can therefore link to other records) but is compared to an initial record where the NHS number is missing and the postcode is different. The initial record itself can be linked to other records that have the same postcode.

HESID, Hospital Episode Statistics ID; NHS, National Health Service.

Table 2 shows the different combinations of identifier values that produced missed matches, among records where sex and date of birth matches—a requirement for HESID at all three steps.

**Table 2:** Scenarios involving different combinations of patient identifiers that resulted in missed matches (where sex and date of birth match)

Sex	Date of birth	NHS number	Hospital	Local ID	Postcode	Missed matches (% of 3609)	
Scenarios where NHS number differs and postcode differs							
1	1	0	1	1	0	840 (23.3)	
1	1	0	1	0	0	43 (1.2)	
1	1	0	0	1	0	33 (0.9)	
1	1	0	0	0	0	316 (8.8)	=1232 (34.1%)
Scenarios where NHS number differs and postcode is the same							
1	1	0	1	1	1	15 (0.4)	
1	1	0	1	0	1	17 (0.5)	
1	1	0	0	0	1	5 (0.1)	=37 (1%)
Other scenarios (see online supplementary table S1)							=2340 (64.8%)

NHS. National Health Service.

Scenarios where postcode differed accounted for the largest proportion (n=1232, 34.1%), but missed matches also occurred when postcode was the same (n=37, 1%). Scenarios where sex or date of birth differed are shown in online supplementary table S1. These all refer to records that do belong to the same patient, but failed to match according to the HESID pseudonymisation algorithm.

Table 3 shows the characteristics of patients experiencing at least one false match (n=115; 0.5% of matched patients) or missed match (n=1554; 1.8% of non-matched patients) within their records, separated according to true match status as defined by the PatientID reference standard.

**Table 3:** Characteristics of linkage error for different patient groups

	Matched patients				Non-matched patients			
	At least one false (n=115)	All correct (n=25 671)	p Value	Total (n=25 786)	At least one missed (n=1554)	All correct (n=84 987)	p Value	Total (n=86 541)
Total	(% of total)				(% of total)			
Age group								
<1 month	14 (0.05)	7749	(ref)	7763	422 (0.49)	13 215	(ref)	13 637
1–12 months	48 (0.19)	8060		8108	509 (0.59)	23 268		23 777
1–4 years	39 (0.15)	4487		4526	315 (0.36)	20 849		21 164
5–10 years	12 (0.05)	2611		2623	177 (0.20)	12 149		12 326
11+ years	2 (0.01)	2764	0.52*	2766	126 (0.15)	15 405	<0.001*	15 531
Missing	0	0	–	0	5 (0.01)	101	0.03	106
Sex								
Male	81 (0.31)	14 711	(ref)	14 792	862 (1%)	47 567	(ref)	48 429
Female	34 (0.13)	10 960	0.01	10 994	674 (0.78%)	37 281	0.96	37 955
Missing	0	0		0	18 (0.02%)	139	<0.001	157
Ethnic group								
White	34 (0.13)	15 150	(ref)	15 184	562 (0.65)	52 590	(ref)	53 152
Mixed	2 (0.01)	576	0.55	578	25 (0.03)	1804	0.93	1829
Asian	10 (0.04)	2340	0.07	2350	89 (0.10)	6394	0.67	6483
Black	11 (0.04)	1021	<0.001	1032	69 (0.08)	3219	0.001	3288
Other	8 (0.03)	579	<0.001	587	114 (0.13)	1905	<0.001	2019
Missing	50 (0.19)	6005	<0.001	6055	695 (0.08)	19 075	<0.001	19 770
Admission								
Planned	42 (0.16)	10 012		10 054	673 (0.78)	32 439	(ref)	33 112
Unplanned	73 (0.28)	15 659	0.59	15 732	881 (1.02)	52 548	<0.001	53 429
Deprivation								
Low	32 (0.12)	7083	(ref)	7115	270 (0.31)	20 314	(ref)	20 584
Middle	38 (0.15)	6861		6899	311 (0.36)	21 194		21 505
High	25 (0.10)	6786	0.48*	6811	254 (0.29)	22 200	0.09*	22 454
Missing†	20 (0.08)	4941	0.62	4961	719 (0.83)	21 279	<0.001	21 998
Provider size								
Small	47 (0.18)	8900	(ref)	8947	535 (0.62)	31 102	(ref)	31 637
Medium	24 (0.09)	7275		7299	606 (0.70)	27 026		27 632
Large	44 (0.17)	9496	0.53*	9540	413 (0.48)	26 859	0.18*	27 272

*p Values for linear trend across groups (univariate).

†Typically due to missing postcode data.

In multivariable logistic regression models (table 4) with a random effect for hospital (allowing for between hospital variation), false matches were more common for younger patients (OR=0.95, 95% CI 0.90 to 0.99), among males (OR=1.77, 95% CI 1.18 to 2.65), in Asian (OR=3.16, 95% CI 1.51 to 6.62), Black (OR=4.12, 95% CI 1.81 to 9.38) and Other ethnic groups (OR=3.23, 95% CI 1.93 to 5.39).

**Table 4:** OR (95% CIs) for patients having at least one false (n=82) or missed (n=2499) match

	At least one false match	p Value	At least one missed match	p Value
	OR (95% CI)		OR (95% CI)	
Age	0.95 (0.90 to 0.99)	0.03	0.93 (0.92 to 0.94)	<0.001
Male	1.77 (1.18 to 2.65)	0.006	0.98 (0.89 to 1.09)	0.75
Unplanned admissions	0.93 (0.63 to 1.38)	0.71	1.03 (0.92 to 1.16)	0.61
Provider medium size (vs small)	0.59 (0.25 to 1.40)	0.23	0.80 (0.41 to 1.58)	0.52
Provider large size (vs small)	0.78 (0.31 to 1.93)	0.59	0.67 (0.30 to 1.49)	0.32
Deprivation=missing (vs low)	1.25 (0.68 to 2.30)	0.48	3.10 (2.65 to 3.62)	<0.001
Deprivation=medium (vs low)	1.26 (0.77 to 2.05)	0.35	0.91 (0.77 to 1.08)	0.30
Deprivation=high (vs low)	0.99 (0.57 to 1.73)	0.98	0.79 (0.66 to 0.95)	0.01
Ethnic group=missing (vs White)	1.25 (0.30 to 5.26)	0.76	0.92 (0.61 to 1.39)	0.69
Ethnic group=Mixed (vs White)	1.56 (0.75 to 3.25)	0.24	1.17 (0.92 to 1.48)	0.20
Ethnic group=Asian (vs White)	3.16 (1.51 to 6.62)	0.02	1.35 (1.03 to 1.77)	0.03
Ethnic group=Black (vs White)	4.12 (1.81 to 9.38)	0.001	3.59 (2.84 to 4.53)	<0.001
Ethnic group=Other (vs White)	3.23 (1.93 to 5.39)	<0.001	2.38 (2.07 to 2.73)	<0.001
	B (95% CI)		B (95% CI)	
Between-hospital variance*	0.75 (0.47 to 1.22)		0.80 (0.61 to 1.04)	

*SD of the hospital-level effect.

Missed matches were less likely to occur per each additional year of age (OR=0.93, 95% CI 0.92 to 0.94). Missed matches were more common however, when postcode and/or socioeconomic deprivation was missing (OR=3.10, 95% 2.65, 3.62) and in Asian (OR=1.35, 95% CI 1.03 to 1.77), Black (OR=3.59, 95% CI 2.84 to 4.53) and Other (OR=2.38, 95% CI 2.07 to 2.73) ethnic groups (table 4).

We found no significant interaction between socioeconomic deprivation and ethnic minority status, and no evidence that the combined effect of living in an area of high deprivation and being in an ethnic minority increased the odds of a missed match (see online supplementary table S2).

In sensitivity analyses, increasing the proportion of dates of birth that were transposed (eg, dd/mm to mm/dd) or NHS numbers that contained typographical errors, increased the missed match and the readmission rate (not shown). Repeating analyses using the most strict validation rule for postcodes (postcode area, postcode district, space, postal sector, unit code) increased the missed match rate to 7.3% without influencing the false match rate of 0.2%. A more relaxed validation rule for postcodes (removing spaces entirely) decreased the missed match rate to 3.8% but increased the false match rate to 3.4%.

Additional analyses using more detailed ethnic groups showed that Black African (OR=2.62, 95% CI 1.32 to 5.23) patients were more likely to experience false matches than White infants, but there were insufficient numbers of Black Carribean or Black Other patients to obtain estimates for these groups. The ethnic group coded as Other comprised Chinese and Other Ethnic categories. Repeating the model for missed matches with these more detailed categories showed a larger and significant effect for the Other Ethnic group (OR=3.19, 95% CI 2.62 to 3.90) than the effect seen for Chinese infants (OR=1.70, 95% CI 0.82 to 3.53).

## Discussion

### Statement of principal findings

Our results are the first evaluation of data linkage errors in hospital administrative records for England. The algorithm has a false match rate of 0.2% and a high missed match rate of 4.1% when applied to paediatric intensive care records, and is particularly sensitive to postcode. The true readmission rate was under-estimated by 3.8%, owing to the high missed match rate. In HES itself, the rates of linkage error are likely to be higher, given that HES has known issues with data quality,^1^ ^4^ ^16^ ^21^ and known variation in the quality of data submitted by providers.^1^ ^4^ ^22^ Additionally, no reference standard data set with patient identifiers currently exists.^2^ Also for the first time, our study highlights that ethnic minority patients (Black, Asian, Other) and patients with missing data in their records, are more affected by data linkage errors.

### Strengths and weaknesses

This study uses a clinical data set from UK hospitals, with the same identifiers available in HES and an independent reference standard patient ID to illustrate the linkage errors that arise when applying the HES linkage and pseudonymisation algorithm used to link national HES data for the NHS in England. Similar identifiers are used in administrative data internationally, suggesting that the scenarios identified will generalise to other settings. Errors in patient identifiers, and missing data, occur even in well-validated data sets such as PICANet and have implications for analysis. In our study, the difference between the true readmission rate and the rate calculated after linkage was 2%, a clinically important and statistically significant underestimation. The emerging literature on data linkage errors has shown that as missed/false matches increase, event rates are underestimated/overestimated respectively.^10^ ^12^ Distorted relative risks can lead to erroneous assessment of relative hospital performance,^23^ and based associations between risk-factors and outcomes, even where overall linkage rates are high.^11^ Linkage errors can also result in clinical harm and breaches of confidentiality.^9^

### Meaning of the study: possible explanations and implications for clinicians and policymakers

Our study was concerned with internal data linkage (ie, linkage between the same individual recorded multiple times within a longitudinal record). When attempts are made to link one data set with another (external data linkage, eg, HES with another administrative data set), linkage success falls to the ‘lowest common denominator’—it depends on the quality of patient identifiers in both data sets. Having access to patient identifiers allowed us to identify specifically that the majority of linkage errors were caused by discrepancies in NHS number and/or postcode (either missing or incorrect). This detailed information provides the opportunity to improve linkage algorithms (eg, by allowing records including missing postcodes to match if other identifiers are the same).

A move from deterministic to probabilistic matching would allow records to match when NHS number differed but all other identifiers were the same.^6^ Allowing records to match based on hospital and local ID would also reduce the missed match rate (see online supplementary table S1), even if the deterministic approach were retained, with little impact on the false match rate. It may also be possible to use prior knowledge about the probability that two records will match in algorithms themselves.^13^ ^24^ Errors in patient identifiers and their impact on data linkage success however, cannot be evaluated without access to patient identifiers and a reference standard. The Personal Demographics Service (PDS) is one possible reference standard for HES, since it purports to be the national demographic database.^25^ Record linkage on a large scale should involve a system of manual review in order to determine linkage quality, which involves checking patient identifiers.^26^

### Unanswered questions and future research

In addition to modifying the algorithm, errors in patient identifiers can be improved by improving data quality at source and with improvement to patient tracing skills^25^ and other mechanisms that generate linkage errors.^4^ Particular problems with accuracy of identifiers for ethnic minority patients might reflect frontline staff being unfamiliar with naming conventions.^27^ We were unable to determine which specific ethnic groups were experiencing the most linkage problems, beyond the broad categories of Black, Asian and Other. Missing data is a further issue, even for mandated fields in HES such as ethnic group.^21^ ^28^ It is important to evaluate and improve data linkage, for the scientific reasons mentioned above but also legal and ethical reasons.^29^ The benefits of linkage of NHS data for service evaluation and research are considered to justify use of patient data without explicit patient consent (Section 251 of the NHS Act 2006), with the HSCIC having legal responsibility for performing the linkage centrally and removing patient identifiers.^29^ However, if linkage results in biased estimates, particularly for certain disadvantaged groups, this justification is undermined.

Proposals to pseudonymise patient identifiers ‘at source’^30^ by local providers, rather than centrally at the HSCIC, could further undermine the justification for linking together hospital care records. Pseudonymisation at source involves identifiers being replaced with a ‘hashed’ identifier before they are released from the provider (whether containing errors or not). This procedure would not address errors in patient identifiers and could increase data linkage error by locking errors in patient identifiers permanently into the data. This would lead to additional biases and prevent manual review that could identify the source of problems, as we have done here.^31^ Although it is possible to use probabilistic matching with pseudonymisation at source,^32^ it is not possible to evaluate errors in identifiers following pseudonymisation at source. This means that there can be no manual review (often considered an important step when using probabilistic matching) and no evaluation of the impact of errors in identifiers on linkage success. Researchers need to collaborate with data providers and those designing data linkage algorithms, in order to reduce errors in patient identifiers, data linkage errors and their harmful consequences.

## References

[1] Academy of Medical Royal Colleges. Hospital episode statistics (HES): improving the quality and value of hospital data. A discussion document. Leeds: Health and Social Care Information Centre, 2011 http://www.hscic.gov.uk/article/1946/Data-quality-resources-for-clinicians

[2] Health and Social Care Information Centre. Replacement of the HES Patient ID (HESID). Leeds: Health and Social Care Information Centre, 2009 http://www.hscic.gov.uk/article/1825/The-processing-cycle-and-HES-data-quality

[3] Health and Social Care Information Centre. A guide to linked mortality data from hospital episode statistics and the office for national statistics. Leeds: Health and Social Care Information Centre, 2013 http://www.hscic.gov.uk/onsmortality

[4] Hagger-Johnson G Identifying possible false matches in anonymised hospital administrative data without patient identifiers. Health Serv Res 2015;50:1162–78. 10.1111/1475-6773.12272 25523215 PMC4545352

[5] Fellegi I, Sunter A A theory for record linkage. J Am Stat Association 1969;64:1183–210. 10.1080/01621459.1969.10501049

[6] Jaro M Probabilistic linkage of large public health data files. Stat Med 1995;14:491–8. 10.1002/sim.4780140510 7792443

[7] Chassin MR, Becher EC The wrong patient. Ann Intern Med 2002;136:826–33. 10.7326/0003-4819-136-11-200206040-00012 12044131

[8] Gray JE, Suresh G, Ursprung R Patient misidentification in the neonatal intensive care unit: quantification of risk. Pediatrics 2006;117:e43–7. 10.1542/peds.2005-0291 16396847

[9] Suresh G, Horbar JD, Plsek P Voluntary anonymous reporting of medical errors for neonatal intensive care. Pediatrics 2004;113:1609–18. 10.1542/peds.113.6.1609 15173481

[10] Schmidlin K, Clough-Gorr K, Spoerri A Impact of unlinked deaths and coding changes on mortality trends in the Swiss National Cohort. BMC Med Inform Decis Mak 2013;13:1 10.1186/1472-6947-13-1 23289362 PMC3547805

[11] Lariscy JT Differential record linkage by Hispanic ethnicity and age in linked mortality studies: implications for the epidemiologic paradox. J Aging Health 2011;23:1263–84. 10.1177/0898264311421369 21934120 PMC4598042

[12] Moore C, Amin J, Gidding H A new method for assessing how sensitivity and specificity of linkage studies affects estimation. PLoS ONE 2014;9:e103690 10.1371/journal.pone.0103690 25068293 PMC4113448

[13] Goldstein H, Harron K, Wade A The analysis of record-linked data using multiple imputation with data value priors. Stat Med 2012;31:3481–93. 10.1002/sim.5508 22807145

[14] Goldstein B, Nadel S The promise and potential of continuous improvement in the pediatric intensive care unit: the evolving story from the United Kingdom Paediatric Intensive Care Audit Network. J Pediatr 2013;163:935–7. 10.1016/j.jpeds.2013.05.044 23810726

[15] Paediatric Intensive Care Audit Network (PICANet). PICANet: a decade of data. Leeds: University of Leeds, 2014 http://www.picanet.org.uk/Audit/Annual-Reporting/

[16] Health and Social Care Information Centre. The HES processing cycle and HES data quality. Leeds: Health and Social Care Information Centre, 2014 http://www.hscic.gov.uk/article/1825/The-processing-cycle-and-HES-data-quality

[17] Health and Social Care Information Centre. Methodology for creation of the HES Patient ID (HESID). Leeds: Health and Social Care Information Centre, 2014 http://www.hscic.gov.uk/article/1825/The-processing-cycle-and-HES-data-quality

[18] Health and Social Care Information Centre. IQAP guidance on unknown, estimated and default birth dates. Leeds: Health and Social Care Information Centre, 2010 http://systems.hscic.gov.uk/data/dataquality/resources

[19] Health and Social Care Information Centre. HES Data dictionary: Inpatients, 2010 http://www.hscic.gov.uk/hesdatadictionary

[20] Department of Communities and Local Government. English indices of deprivation 2010. London: Stationery Office, 2011 https://www.gov.uk/government/statistics/english-indices-of-deprivation-2010

[21] Dattani N, Datta-Nemdharry P, Macfarlane A Linking maternity data for England 2007: methods and data quality (ONS Report). London: Office of National Statistics, 2007 http://www.ons.gov.uk/ons/rel/hsq/health-statistics-quarterly/no--53--spring-2012/linkage-of-maternity-hospital-episode-statistics-data.html 10.1057/hsq.2011.3 21372845

[22] Royal College of Obstetricians and Gynaecologists. Patterns of maternity care in English NHS hospitals. London: RCOG, 2013 https://www.rcog.org.uk/en/guidelines-research-services/audit-quality-improvement/clinical-indicators-project/

[23] Fraser J, Maskrey C, Taylor H Evaluation of the Paediatric Index of Mortality in children managed on adult intensive care units. Arch Dis Child 2004;89:974–6. 10.1136/adc.2003.037614 15383445 PMC1719689

[24] Harron K, Wade A, Gilbert R Calculating matching probabilities in record linkage. BMC Med Res Methodol 2014;14:36 10.1186/1471-2288-14-36 24597489 PMC4015706

[25] Connecting for Health. PDS tracing guidance for front line NHS staff. Leeds: Health and Social Care Information Centre, 2005 http://webarchive.nationalarchives.gov.uk/20130502102046/http://www.connectingforhealth.nhs.uk/elearning/pds/flash/supporting%20documents/PDS%20Tracing%20Guidance%20v1.0.pdf

[26] Barry S, Dinnett E, Kean S Are routinely collected NHS administrative records suitable for endpoint identification in clinical trials? Evidence from the West of Scotland Coronary Prevention Study. PLoS ONE 2013;8:e75379 10.1371/journal.pone.0075379 24058681 PMC3772901

[27] Health and Social Care Information Centre. IQAP guidance on ethnic naming conventions. Leeds: Health and Social Care Information Centre, 2009 http://systems.hscic.gov.uk/data/dataquality/resources/dqm002408.pdf

[28] Health and Social Care Information Centre. How good is HES ethnic coding and where do the problems lie? Leeds: NHS Information Centre, 2011 http://www.hscic.gov.uk/hes

[29] Secretary of State. Health and Social Care Act 2012. London: The Stationary Office, 2010 http://www.legislation.gov.uk/ukpga/2012/7/contents/enacted

[30] Parry J Pseudonymised data can protect patient confidentiality. Secondary pseudonymised data can protect patient confidentiality, 2014 http://www.hsj.co.uk/resource-centre/supplements/pseudonymised-data-can-protect-patient-confidentiality/5071244.article

[31] Office of National Statistics. Beyond 2011: matching anonymous data. London: ONS, 2013 http://www.ons.gov.uk/ons/about-ons/who-ons-are/programmes-and-projects/beyond-2011/reports-and-publications/methods-and-policies-reports/index.html

[32] Schnell R, Bachteler T, Reiher Jr Privacy-preserving record linkage using Bloom filters. BMC Med Inform Decis Mak 2009;9:41 10.1186/1472-6947-9-41 19706187 PMC2753305

